# Butein inhibits ethanol-induced activation of liver stellate cells through TGF-β, NFκB, p38, and JNK signaling pathways and inhibition of oxidative stress

**DOI:** 10.1007/s00535-012-0619-7

**Published:** 2012-06-22

**Authors:** Agnieszka Szuster-Ciesielska, Magdalena Mizerska-Dudka, Jadwiga Daniluk, Martyna Kandefer-Szerszeń

**Affiliations:** 1 Department of Virology and Immunology, Maria Curie-Skłodowska University, Akademicka 19, 20-033 Lublin, Poland; 2 Department and Clinic of Gastroenterology, Medical University, Jaczewskiego 8, 20-950 Lublin, Poland

**Keywords:** Hepatic stellate cells, HepG2, Butein, Cytokines, MAPK, NFκB

## Abstract

**Background:**

Butein has been reported to prevent and partly reverse liver fibrosis in vivo; however, the mechanisms of its action are poorly understood. We, therefore, aimed to determine the antifibrotic potential of butein.

**Methods:**

We assessed the influence of the incubation of hepatic stellate cells (HSCs) and hepatoma cells (HepG2) with butein on sensitivity to ethanol- or acetaldehyde-induced toxicity; the production of reactive oxygen species (ROS); the expression of markers of HSC activation, including smooth muscle α-actin (α-SMA) and procollagen I; and the production of transforming growth factor-β1 (TGF-β1), metalloproteinases-2 and -13 (MMP-2and MMP-13), and tissue inhibitors of metalloproteinases (TIMPs). The influence of butein on intracellular signals in HSCs; i.e., nuclear factor-κB (NFκB), c-Jun N-terminal kinase (JNK), and p38 mitogen-activated protein kinase (p38 MAPK) induced by ethanol was estimated.

**Results:**

Butein protected HSCs and HepG2 cells against ethanol toxicity by the inhibition of ethanol- or acetaldehyde-induced production of ROS when cells were incubated separately or in co-cultures; butein also inhibited HSC activation measured as the production of α-SMA and procollagen I. As well, butein downregulated ethanol- or acetaldehyde-induced HSC migration and the production of TGF-β, TIMP-1, and TIMP-2; decreased the activity of MMP-2; and increased the activity of MMP-13. In ethanol-induced HSCs, butein inhibited the activation of the p38 MAPK and JNK transduction pathways as well as significantly inhibiting the phosphorylation of NF κB inhibitor (IκB) and Smad3.

**Conclusions:**

The results indicated that butein inhibited ethanol- and acetaldehyde-induced activation of HSCs at different levels, acting as an antioxidant and inhibitor of ethanol-induced MAPK, TGF-β, and NFκB/IκB transduction signaling; this result makes butein a promising agent for antifibrotic therapies.

**Electronic supplementary material:**

The online version of this article (doi:10.1007/s00535-012-0619-7) contains supplementary material, which is available to authorized users.

## Introduction

Liver fibrosis is caused by a variety of agents, including chronic viral hepatitis, alcohol toxicity, autoimmune disease, and hereditary metabolic disorders. For all of these diseases there is a common pathologic mechanism that leads to fibrosis: the generation and proliferation of smooth muscle α-actin (α-SMA)-positive myofibroblasts of periportal and perisinusoidal origin which arise as a consequence of the activation of hepatic stellate cells (HSCs). HSCs exist in the normal liver as quiescent retinoid-storing cells, which, in response to injury, are activated to become proliferative, profibrogenic cells [1–3]. The activated HSCs are a rich source of type I and III fibrillar collagen and also secrete high levels of tissue inhibitors of metalloproteinase (TIMPs) [4]. Several soluble factors, including growth factors, cytokines, chemokines, and oxidative stress products, derived from hepatocytes, play a role in the activation of HSCs. The activation of HSCs is associated with the sequential expression of several key cytokines and their surface receptors, including transforming growth factor-β (TGF-β) and its receptors [5]. Endogenous expression of TGF-β in the liver induces liver fibrosis, and the blockade of TGF-β signaling by multiple methods prevents the progression of liver fibrosis in experimental animals [6]. TGF-β downstream signaling is mediated by Smad2 and Smad3, which are structurally similar but functionally distinct. They are differentially activated by TGF-β in quiescent and activated HSCs and play different roles in HSC function [7, 8].

The development of liver fibrosis in alcoholics has been linked to the oxidation of ethanol to the highly reactive compound acetaldehyde. At concentrations that have been detected in hepatic venous blood after alcohol consumption, acetaldehyde stimulated type I collagen synthesis and gene transcription in cultured rat and human HSCs through protein kinase C (PKC) activation [9]. Acetaldehyde was also shown to increase nuclear factor-κB (NFκB) (p65) and its binding to the α_2_(I) collagen promoter [10] and to enhance NFκB by a mechanism dependent on the accumulation of H_2_O_2_ [11–13]. CYP2E1 is an important source of reactive oxygen species (ROS) in alcohol-induced injury and fibrosis, as it generates superoxide (O_2_^−^) and hydrogen peroxide (H_2_O_2_). It has been reported that the inhibition of CYP2E1 activity by diallylsulfide (DAS) prevented the induction of collagen I gene expression in rat stellate cells overexpressing CYP2E1 [14]. Oxidative stress also activates c-Jun N-terminal kinase (JNK), a protein which regulates the secretion of proinflammatory cytokines by cultured HSCs [15, 16].

Matrix metalloproteinases (MMPs), a family of zinc metallo-endopeptidases, are promptly expressed by HSCs in response to diverse hepatic toxins. In vitro experiments have demonstrated the role of MMPs in the activation of HSCs. Also, the proliferation of HSCs is promoted by pericellular collagen I proteolysis acting via αvβ3 integrins [17]. Conversely, MMPs may also contribute to the regression of liver fibrosis through cleavage of the fibrillar extracellular matrix (ECM) and the promotion of apoptosis in the activated HSCs. Thus MMPs play a dual role in liver fibrosis, depending on the timing of their production [18].

To prevent the progression of hepatic fibrosis, various types of compounds that interfere with HSC proliferation and activation have been developed as antifibrogenic agents. Among others, butein (3,4,2′,4′-tetrahydroxychalcone), a polyphenolic compound extracted from the stem bark of cashews and *Rhus verniciflua* Stokes has been shown to suppress liver fibrosis induced by carbon tetrachloride [19] and to inhibit myofibroblastic differentiation of rat HSCs [20]. Its derivative, with improved bioavailability, has been shown to have a potent antiproliferative effect mediated by the activation of ERK, with ERK activation leading to the transcriptional activation of AP-1 and, consequently, to heme oxygenase 1 expression in hepatic stellate cells [21]. However, butein also exhibits anti-inflammatory and antitumor effects through the activation of other pathways, such as ERK 1/2 and NF-κB signaling [21–23].

The aim of this study was to investigate the effect of butein on the activation of rat HSCs cultured in vitro. To assess the mechanisms of butein’s influence on HSC activation, we examined whether butein changed the sensitivity of hepatocytes and HSCs to ethanol cytotoxicity, and whether it changed the production of ROS in hepatocytes and HSCs. We also examined whether butein influenced the production of TGF-β, MMPs, and TIMPs in ethanol- and acetaldehyde-activated HSCs. In activated HSCs we examined the influence of butein on intracellular signaling, such as TGF-β-induced signaling, and NFκB, JNK, and p38 MAPK activation. Studies were performed with a well-characterized HSC clone (CFSC-2G cell line) as a model to investigate HSC activation; data from this model are comparable to the data obtained from in vivo animal models, as well as human samples [24]. The CFSC-2G cell line has a phenotype similar to that of freshly isolated HSCs [25]. Additionally, in some experiments we also used HepG2 cells to study the effect of butein in co-cultures of HSCs with hepatocytes.

## Materials and methods

### Cell cultures

A rat HSC cell line, CFSC-2G, was kindly provided by Dr. Marcos Rojkind (Department of Clinical Investigation, Walter Reed Army Medical Center, Washington, DC, USA). HSCs were cultured in Eagle’s medium (MEM), supplemented with 5 % heat-inactivated fetal calf serum (FCS), 1 % nonessential amino acids (NEAA), and 1 % antibiotic-antimycotic, pH 7.4. The cells were seeded in tissue culture plates (Falcon, Bedford, MA, USA) and incubated at 37 °C in a humidified atmosphere of 5 % CO_2_. Cells were subcultured twice a week by trypsinization in a 0.25 % trypsin–ethylenediamine tetraacetic acid (EDTA) solution after washing with Ca–Mg-free saline. This non-tumoral cell line is characterized by low basal levels of type I collagen gene expression and by the presence of mRNA for α-SMA; hence, in all experiments we starved these cells by MEM supplementation with only 0.1 % FCS. The human hepatoma HepG2 cell line retains many hepatocyte functions and was obtained from the American Type Culture Collection (Manassas, VA, USA). These cells were cultured in Eagle’s medium (MEM), supplemented with 10 % heat-inactivated FCS, 2 mM l-glutamine, 1 % NEAA, 1.5 g/l sodium bicarbonate, and 1 % antibiotic-antimycotic, pH 7.4. The cells were seeded in tissue culture plates (Falcon) and incubated at 37 °C in a humidified atmosphere with 5 % CO_2_. HepG2 cells were subcultured twice a week by trypsinization in 0.25 % trypsin–EDTA solution after washing with Ca–Mg-free saline. The culture media and antibiotics were purchased from Gibco (Grand Island, NY, USA), and 0.25 % trypsin–EDTA, FCS, and NEAA were obtained from Sigma-Aldrich (Steinheim, Germany). In some experiments, Hanks’ balanced salt solution (HBSS) (Sigma-Aldrich) was used.

### The influence of butein on the viability of HSCs and HepG2 cells treated with ethanol or acetaldehyde as the ethanol metabolite

In preliminary experiments (data not shown) on the influence of butein on cell viability and proliferation we detected that 1–10 μM butein exhibited no toxicity and did not significantly influence the proliferation of CFSC-2G or HepG2 cells after 24-h incubation. Therefore, for further experiments 1 and 10 μM butein was used. HepG2 cells were grown in 96-well plastic plates (Nunc, Roskilde, Denmark), at 4 × 10^4^ cells/well. After 24-h incubation, the medium was replaced with a fresh one with the addition of 2 % FCS and 1 μM or 10 μM butein (Sigma-Aldrich). HSCs were grown in 96-well plastic plates (Nunc) at a density of 2 × 10^4^ cells/well in Eagle’s medium (MEM) supplemented with 5 % FCS. After 24-h incubation, the medium was replaced with a fresh one with the addition of 0.1 % FCS and 1 or 10 μM butein (Sigma-Aldrich). After another 24 h of incubation, different ethanol (5–100 mM for CFSC-2G cells and 5–50 mM for HepG2 cells) and acetaldehyde concentrations (75–500 μM for CFSC-2G and 75–175 μM for HepG2 cells) were added. It should be noted that these ethanol and acetaldehyde concentrations were chosen in preliminary experiments, in which it was detected that HepG2 cells were several times more sensitive to ethanol and acetaldehyde toxicity than CSFC-2G. Ethanol and acetaldehyde were purchased from Merck (Darmstadt, Germany) and maintained as 1 M stock solutions. The cells treated with ethanol or acetaldehyde were maintained in closed-lid containers in a humidified CO_2_-incubator at 37 °C for 24 h. The toxicity of these chemicals was determined by 3-(4,5-dimethylthiazo-2-yl)-2,5-diphenyl-tetrazolium bromide (MTT) assay, in which the yellow tetrazolium salt was metabolized by viable cells to purple formazan crystals. The HSCs were incubated for 3 h with the MTT solution (5 mg/ml). Formazan crystals were solubilized overnight in sodium dodecylsulfate (SDS) buffer (10 % SDS in 0.01 N HCl, Sigma-Aldrich), and the product was quantified spectrophotometrically by measuring absorbance at a wavelength of 570 nm, using an E-max Microplate Reader (Molecular Devices, Menlo Park, CA, USA). The data are presented as percentages of control cell viability from four independent experiments each with eight separate cell cultures.

### Measurement of superoxide anion (O_2_^−^) production by cytochrome c reduction assay [26]

HepG2 cells were grown in 96-well plastic plates (4 × 10^4^ cells/well) and HSCs were grown in 96-well plastic plates (2 × 10^4^ cells/well) for 24 h at 37 °C in a humidified atmosphere of 5 % CO_2_. Then, the cultures were washed twice with HBSS, and the culture medium was replaced with fresh 0.1 % FCS-MEM (CFSC-2G cells) or with 2 % FCS-MEM (HepG2 cells) with or without 10 μM butein. The next day, an assay for superoxide anion was performed. Briefly, HBSS (207.5 μl), 12.5 μl of cytochrome c solution in HBSS (to achieve a final concentration of 75 μM), 5 μl of either superoxide dismutase (SOD) solution (to achieve a final concentration of 60 U/ml) or 5 μl HBSS, and 25 μl ethanol solution in HBSS (final concentration of 5, 10, or 50 mM) or acetaldehyde (final concentration of 75 or 175 μM) were added to each well on a 96-well plate. Also, control wells were used in which cells were incubated without ethanol or acetaldehyde. The microplate was incubated at 37 °C for 60 min and transferred to a microplate reader. The absorbance values at 550 nm (the differences in optical density [OD] between samples with and without SOD) were converted to nanomoles of O_2_^−^ based on the extinction coefficient of cytochrome c: Δ*E*_550_ = 21 × 10^3 ^M^−1 ^cm^−1^. The results were expressed as nanomoles of O_2_^−^ per 1 × 10^6^ cells per 60 min. Four independent experiments were repeated, each with eight separate cell cultures. All chemicals were purchased from Sigma-Aldrich.

### Migration assay

#### Cell migration was assessed using an in vitro wound closure assay

HSCs were plated at a density of 3 × 10^5^ cells/ml on 4-cm culture dishes (Nunc) (2 ml/dish) in 5 % FCS-MEM for 24 h. Then, one linear wound was scraped in each well with a sterile pipette tip (P300). The wounded monolayers were rinsed twice with culture medium to remove all cellular debris, and the medium was replaced with a fresh one with the addition of 0.1 % FCS-MEM and, in some cultures, also 10 μM butein. After 24-h incubation, 50 mM ethanol or 175 μM acetaldehyde was added to the wells with or without butein. Control cells were cultured in 0.1 % FCS-MEM. The number of cells which had migrated into the wounded area after 24 h was estimated in the control and in the cultures treated with ethanol that were pretreated or not with 10 μM butein. Plates were stained by the May–Grünwald–Giemsa method. The observation was performed under an Olympus BX51 System Microscope (Olympus Optical, Tokyo, Japan), and micrographs were prepared using analySIS software (Soft Imaging System, Münster, Germany). Cells which had migrated to the wounded areas were counted on micrographs, and the results were expressed as the mean number of cells which had migrated to 100 selected wounded areas taken from three micrographs.

#### Treatment of HSCs with ethanol and 10 μM butein

HSCs were grown in 6-well plastic plates (2 × 10^5^ cells/ml, 5 ml/well) in 5 % FCS-MEM for 24 h in a humidified CO_2_-incubator at 37 °C. Then, the medium was replaced with fresh 0.1 % FCS-MEM with or without 10 μM butein, and the cells were incubated for another 24 h at 37 °C. After that, the inducer, ethanol, at different concentrations (final concentration 5, 10 or 50 mM), was added to the medium (in some experiments 175 μM acetaldehyde was also used). To exclude only the preincubation effect, in another experiment HSCs were treated with ethanol first and after 24-h incubation with butein 10 μM, and parameters of cell activation (such as the production of α-SMA and procollagen I) were measured by western blot. Plates were prepared in duplicate:

- after 20 min of incubation and washing twice with phosphate-buffered saline (PBS), the cells were collected for western blot analysis of phospho- and total NFκB, phospho- and total IκB, phospho- and total JNK, and phospho- and total p38 MAPK.
- after 24 h of incubation, the cultures were washed twice with PBS, and the cells were harvested for western blot analysis of α-SMA, procollagen I, TIMP-1, TIMP-2, MMP-13, and phospho- and total Smad3.

Additionally the cell culture supernatants were centrifuged and frozen immediately at −80 °C for further cytokine (TGF-β), MMP-2, and TIMP-1 level measurements, using a sandwich enzyme-linked immunosorbent assay (ELISA) according to the manufacturer’s instructions; ELISA kits for the detection of rat proteins were purchased from R&D Systems. All experiments were performed three times each with four separate cell cultures for one sample.

#### Co-cultures of HepG2 cells activated with ethanol or acetaldehyde with HSCs

HSCs at the density of 2 × 10^5^/ml in MEM medium supplemented with 5 % of FCS were seeded in 6-well plastic plates. At the same time, HepG2 cells at the density of 1 × 10^6^/ml were seeded into tissue culture inserts with a membrane (pore diameter 0.4 μm) and incubated for 24 h at 37 °C. After that the HepG2 cells in the inserts were treated with ethanol (final concentration 50 mM) or acetaldehyde (final concentration 175 μM) diluted in MEM supplemented with 0.1 % of FCS for 3 h at 37 °C, washed, and moved into the cultures of stellate cells in the wells of plastic plates in which the medium was supplemented with antioxidant enzymes such as SOD (240 U/ml) and catalase (CAT; 40 U/ml). Appropriate controls were also prepared. Cells were co-cultured for 24 h at 37 °C and HSCs were collected for the measurements of α-SMA and procollagen I expression. Also, cell co-culture supernatants were collected for further TGF-β measurement. Experiments were done in triplicate (each with three separate cell cultures).

#### Western blot analysis

HSCs were harvested and lysed in RIPA buffer (50 mM Tris/HCl pH 7.4, 150 mM NaCl, 1 % Triton X-100, 1 mM EDTA, 1 % sodium deoxycholate, 0.1 % SDS, 1 mM Na_3_VO_4_, 10 mM NaF, and protease inhibitor cocktail), and then centrifuged at 10,000 rpm/5 min at 4 °C. Proteins were assayed using a BCA Protein Assay Kit (Pierce, Rockford, IL, USA). For western blot analysis, supernatants of RIPA cell lysates were solubilized in 5×SDS sample buffer (100 mM Tris/HCl pH 6.8, 25 % glycerol, 2 % SDS, 0.01 % bromophenol blue, 3 % β-mercaptoethanol) and then boiled for 5 min at 100 °C. Equal amounts of the total cellular protein extract were separated on 10 % SDS-polyacrylamide gel electrophoresis (PAGE) at 200 V for 1 h under reducing conditions and electrotransferred in a semi-dry way to polyvinylidine difluoride membranes (PVDF; Millipore, Whatman) at 15 V for 15 min in a transfer buffer, pH 8.1 (47.8 mM Tris/HCl, 0.293 % glycine, 20 % methanol). After blocking for 15 min at room temperature with 10 % dried nonfat milk/TBS/0.1 % Tween 20, the membranes were probed overnight at +4 °C with primary antibodies (diluted in 1 % bovine serum albumin [BSA]/TBS/0.1 % Tween 20) as follows: rabbit polyclonal anti-procollagen type I (1:250; Santa Cruz Biotechnology, Santa Cruz, CA, USA), mouse monoclonal anti-α-SMA (1:1000; Sigma-Aldrich), mouse monoclonal anti-β-actin (1:4000; Sigma-Aldrich), mouse monoclonal anti-TIMP-1 (1:500; R&D Systems), rabbit monoclonal anti-TIMP-2 (1:1000; Sigma-Aldrich), rabbit polyclonal anti-MMP-13 (1:200; USBiological), rabbit monoclonal anti-total Smad3 (1:2000; Epitomics), rabbit monoclonal anti-phospho Smad3 (1:1000; Epitomics), rabbit polyclonal anti-total NFκB p65 (1:2000; Chemicon), rabbit polyclonal anti-phospho NFκB p65 (1:500; Rockland), rabbit polyclonal anti-total IκB-α (1:2000; Sigma-Aldrich), mouse monoclonal anti-phospho IκB-α (1:1000; USBiological), rabbit polyclonal anti-total JNK (1:2000; Sigma-Aldrich), rabbit polyclonal anti-phospho JNK 1/2 (1:1000; Sigma-Aldrich), rabbit polyclonal anti-total p38 (1: 10000; Sigma-Aldrich), and rabbit polyclonal anti-phospho p38 (1:1000; Sigma-Aldrich). After repeated washing (TBS/0.1 % Tween 20), the membranes were incubated with a horseradish peroxidase-conjugated secondary (anti-rabbit or anti-mouse) antibody (1:4000, in 1 % BSA/TBS/0.1 % Tween 20; Amersham Bioscience, Buckinghamshire, UK), and visualized using an enhanced chemiluminescence reaction (ECL Western Blotting System; Amersham Bioscence). Protein bands were scanned, and the band intensities were quantified using ImageJ densitometry software.

### Statistical analysis

Values are expressed as means ± SD. The significance of differences was determined with the use of analysis of variance (ANOVA) (Statistica computer package). A number of statistical tests were used; these included a two-way ANOVA test with post-hoc Tukey’s test and Wilcoxon’s paired test for comparisons inside groups. *P* values of ≤0.05 were considered to be significant.

## Results

### Butein attenuates ethanol- and acetaldehyde-induced cytotoxicity in HSCs and HepG2 cells

**Fig. 1a-d:**
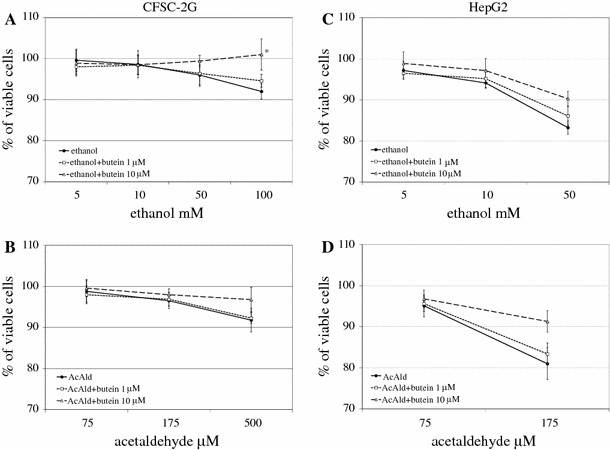
The influence of butein on ethanol- and acetaldehyde-induced toxicity in hepatic stellate cells (HSCs; *CFSC-2G*) and HepG2 cells. Cells were preincubated in medium with 1 or 10 μM butein for 24 h. Subsequently, ethanol or acetaldehyde at the indicated concentrations was added. After 24 h of incubation, the toxicity was determined by the MTT method. Values are means ± SD of results from four independent experiments each with eight separate cell cultures. *Statistically significant at *p* ≤ 0.05 in comparison to cells incubated with ethanol alone (Wilcoxon test)

In earlier experiments (data not shown) on the influence of butein on cell viability and proliferation, we detected that 1–10 μM butein exhibited no toxicity and did not significantly influence CFSC-2G and HepG2 cell proliferation after 24-h incubation. Therefore, for further experiments 1 and 10 μM butein was used. As can be seen from Fig. 1, preincubation of HSCs with 10 μM butein attenuated the toxicity of ethanol and acetaldehyde more effectively than 1 μM, especially when the highest concentrations of ethanol and acetaldehyde were used; hence, we decided to apply 10 μM butein in further experiments. It should be stressed that we detected that HSCs were more resistant than HepG2 cells to the toxic effect of ethanol and acetaldehyde; therefore, in our experiments higher ethanol and acetaldehyde concentrations were used to treat HSCs than HepG2 cells.

### The influence of butein on reactive oxygen species (ROS) production in ethanol- or acetaldehyde-treated HSCs and HepG2 cells

**Fig. 2:**
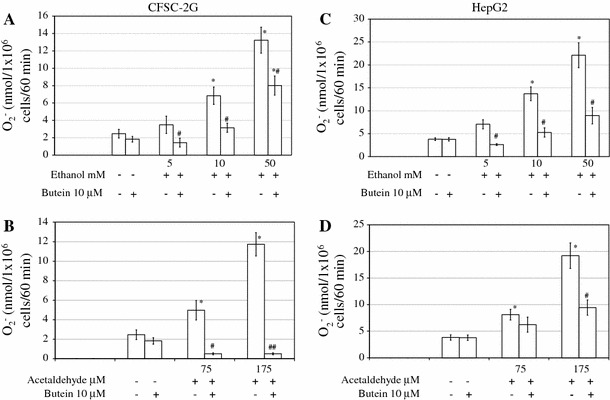
Preincubation of HSCs and HepG2 cells for 24 h with 10 μM butein inhibits ethanol-induced (**a**, **c**) and acetaldehyde-induced (**b**, **d**) superoxide anion production. Cells were preincubated with butein for 24 h, after which an assay for superoxide anion was performed in which nanomoles of O_2_^−^ per 1 × 10^6^ cells per 60 min were calculated. Results are expressed as means ± SD of four independent experiments each with eight separate cell cultures. *Significantly different from respective controls (cells incubated without ethanol, acetaldehyde, and butein or treated only with butein), *p* ≤ 0.05. ^#^Statistically significant at *p* ≤ 0.05 in comparison to cells treated with ethanol or acetaldehyde alone. Butein significantly changed both the ethanol and acetaldehyde effects, *p* ≤ 0.01 (two-way analysis of variance [ANOVA])

Ethanol and its metabolite acetaldehyde are known as strong inducers of superoxide anion production in HSCs and HepG2 cells (Fig. 2). When HSCs and HepG2 cells were preincubated for 24 h with 10 μM butein and then ethanol or acetaldehyde was used as an inducer of “oxidative burst”, butein significantly inhibited the superoxide anion production induced by ethanol. In regard to acetaldehyde, when CFSC-2G cells were preincubated with butein the inhibition of superoxide anion production was stronger than that in HepG2 cells. Butein alone did not induce O_2_^−^ production.

### The influence of butein supplementation on ethanol-induced α-SMA and procollagen I production. Butein induces HSC quiescence

**Fig. 3:**
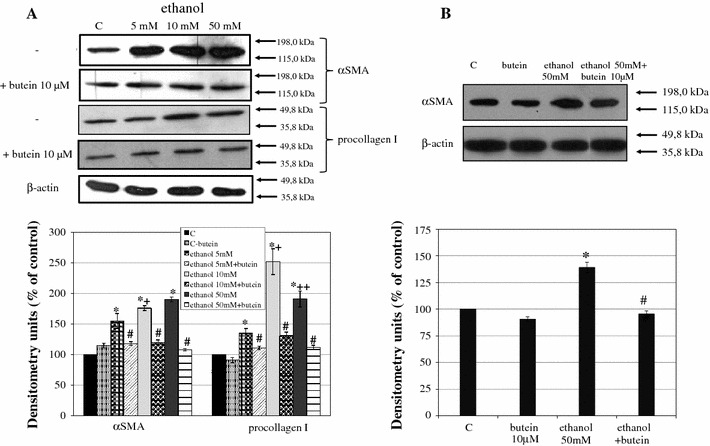
Incubation of HSCs with 10 μM butein induces quiescence of cells activated by ethanol. HSCs were preincubated with butein for 24 h before treatment with ethanol (**a**) or first activated by ethanol for 24 h and incubated with butein for the next 24 h (**b**; lane *C* control). Markers of HSC activation such as α-smooth muscle actin (*α-SMA*) and procollagen I were measured by western blot. β-Actin expression served as the loading control. On the *right*, the *arrows* indicate the position of the molecular weight markers used in the experiments. Representative blots are shown. Experiments were done in triplicate (each with four separate cell cultures), and the bars represent means ± SD. *Significantly different from respective control (cells incubated without ethanol); *p* ≤ 0.05. ^#^Statistically significant difference, at *p* ≤ 0.05, in comparison to cells treated with ethanol alone. ^+^Significantly different from the lower ethanol concentration (5 mM), ^++^(10 mM); *p* ≤ 0.01. Butein significantly changed the ethanol effect, *p* ≤ 0.01 (two-way ANOVA)

HSCs that were starved by incubation of the cells in a medium with 0.1 % FCS were subsequently incubated with ethanol. The intracellular levels of α-SMA and procollagen type I were estimated by western blot. Ethanol induced the production of both α-SMA and procollagen type I in a concentration-dependent manner (Fig. 3). Preincubation with 10 μM butein and subsequent activation by different concentrations of ethanol significantly inhibited the expression of the markers of HSC activation (Fig. 3a). Moreover, butein was active not only in protecting cells against activation but also was effective after activation. When HSCs were activated by 50 mM ethanol for 24 h and subsequently incubated with 10 μM butein, inhibition of their activation was also detected (Fig. 3b).

### Butein induces HSC quiescence in co-cultures of HSCs with ethanol-activated HepG2 cells

**Fig. 4:**
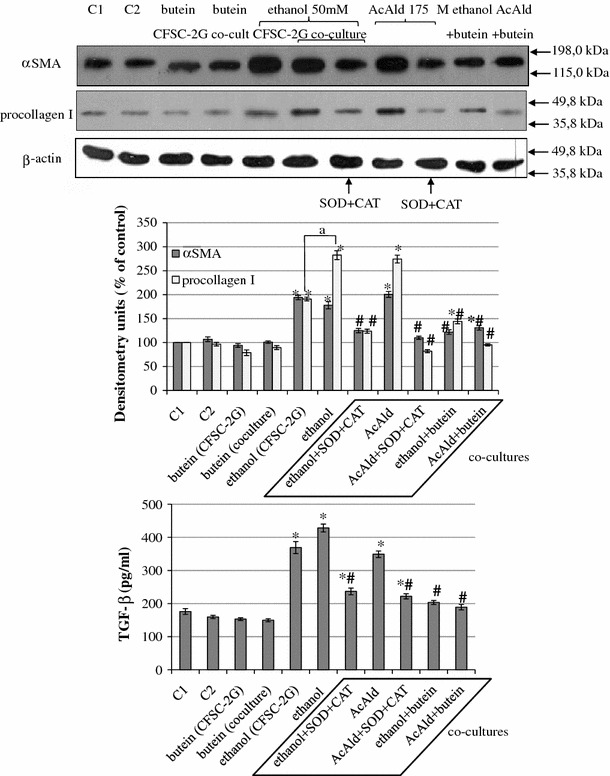
Butein inhibits HSC activation stimulated by co-cultures of HSCs with HepG2 cells treated with ethanol or acetaldehyde. HSCs were seeded in 6-well plastic plates. At the same time HepG2 cells were seeded into tissue culture inserts with a membrane (pore diameter 0.4 μm) and incubated for 24 h at 37 °C. After that, HepG2 cells in the inserts were treated with ethanol or acetaldehyde for 3 h at 37 °C, washed, and moved into cultures of stellate cells in the wells of plastic plates in which the medium was supplemented with antioxidant enzymes such as superoxide dismutase (*SOD*) (240 U/ml) and catalase (*CAT*) (40 U/ml). Appropriate controls were also prepared (*C1* HSCs only, *C2* HSCs with HepG2 cells without any chemicals). Cells were co-cultured for 24 h at 37 °C and HSCs were collected for the measurement of α-SMA and procollagen I expression. In co-culture supernatants transforming growth factor-β (*TGF-β*) was also determined (enzyme-linked immunosorbent assay [ELISA] method). Experiments were done in triplicate (each with three separate cell cultures), and the bars represent means ± SD. *Significantly different from respective controls (cells incubated without ethanol or acetaldehyde); *p* ≤ 0.05. ^#^Statistically significant at *p* ≤ 0.05 in comparison to co-culture where HepG2 cells had been treated with ethanol or acetaldehyde alone. ^a^Significantly different in comparison to ethanol treatment in CFSC-2G only, *p* ≤ 0.05. Butein significantly changed the ethanol or acetaldehyde effect, *p* ≤ 0.01 (two-way ANOVA)

HSCs were seeded in 6-well plastic plates and co-cultured with HepG2 cells seeded into tissue culture inserts with a membrane. HepG2 cells in the inserts were treated with ethanol or acetaldehyde diluted in MEM supplemented with 0.1 % of FCS for 3 h at 37 °C, washed, and moved into cultures of HSCs in which the medium was supplemented with antioxidant enzymes such as SOD (240 U/ml) and CAT (40 U/ml). The cells were co-cultured for 24 h at 37 °C and HSCs were collected for the measurement of α-SMA and procollagen I expression. As can be seen from Fig. 4, treatment of HepG2 cells with ethanol or acetaldehyde and co-culture of these cells with HSCs caused enhanced expression of α-SMA and procollagen I in the HSCs, which could be attenuated by the addition of antioxidant enzymes such as SOD and CAT or the addition of butein. In contrast to the production of α-SMA, the production of procollagen in the co-cultures in which HepG2 cells had been activated with ethanol was significantly higher than that in HSCs alone. The inhibitory effect of butein was comparable to the effect of the antioxidant enzymes, suggesting that the ROS generated by HepG2 cells play a major role in the activation of HSCs.

### The influence of butein on HSC motility

**Fig. 5:**
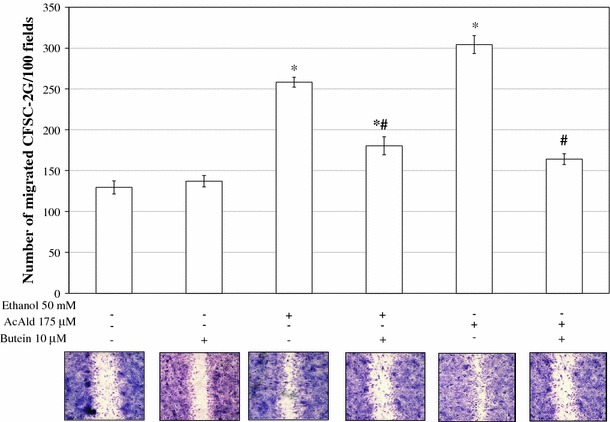
Butein inhibits motility of HSCs. A wound healing assay was performed on HSCs grown to a confluent cell layer in which a wound was scraped to remove a linear area of cells. The cultures were treated with 10 μM butein for 24 h and then 50 mM ethanol or 175 μM acetaldehyde was added, and the cells were allowed 24 h to migrate. Representative images of different conditions are shown. The experiment was repeated five times. *Statistically significant at *p* ≤ 0.05 in comparison to respective controls (cells not treated or treated only with butein). ^#^Statistically significant at *p* ≤ 0.05 in comparison to cells treated with ethanol or acetaldehyde alone (Wilcoxon test)

When the migration of HSCs was examined by the wound closure assay (Fig. 5), the addition of butein to the incubation medium significantly inhibited the ethanol- or acetaldehyde-induced migration of cells in the area of the linear wound scraped in the monolayer of HSCs.

### Butein downregulates TGF-β1, MMP-2, TIMP-1, and TIMP-2 production in ethanol- or acetaldehyde-activated HSCs

**Fig. 6a, b:**
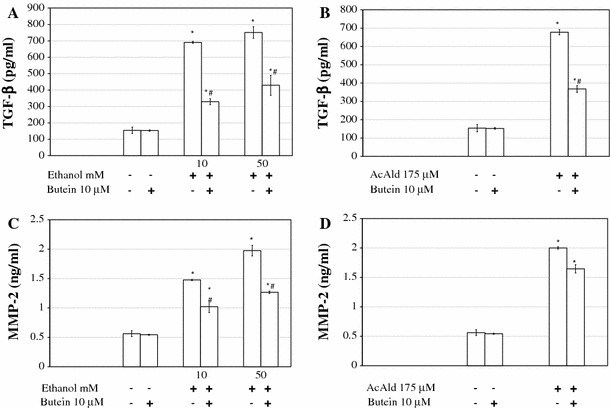
Preincubation of HSCs with 10 μM butein inhibits ethanol- and acetaldehyde-induced production of TGF-β and matrix metalloproteinase-2 (*MMP-2*). The cells were preincubated with 10 μM butein for 24 h and subsequently induced to produce TGF-β and MMP-2 by the addition of ethanol at the indicated concentrations or by the addition of 175 μM acetaldehyde. The levels of TGF-β and MMP-2 were measured by ELISA and are shown as the means ± SD of three independent experiments each with four separate cell cultures. *Significantly different from respective controls (cells not treated or treated only with butein), *p* ≤ 0.01. ^#^Statistically significant at *p* ≤ 0.05 in comparison to cells treated with ethanol or acetaldehyde alone. Butein significantly changed the ethanol or acetaldehyde effect, *p* ≤ 0.01 (two-way ANOVA)

**Fig. 7:**
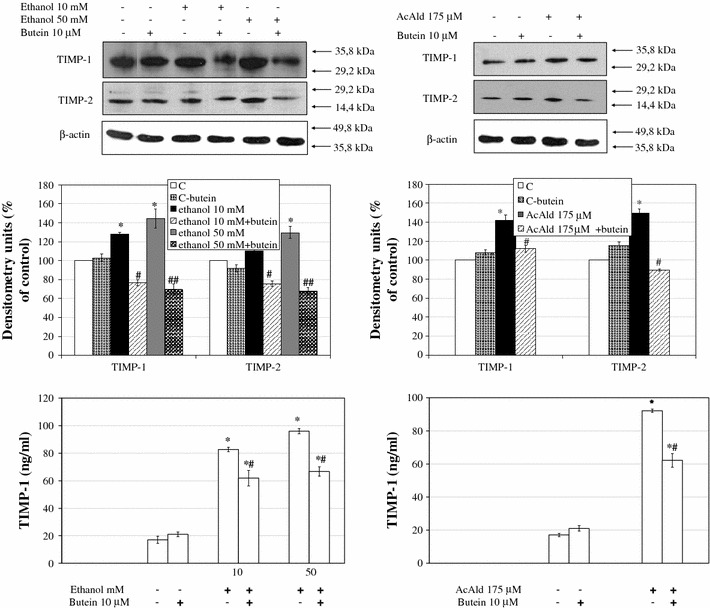
The effect of preincubation of HSCs with 10 μM butein on parameters related to extracellular matrix remodeling induced by ethanol. Western blot analyses for tissue inhibitor of metalloproteinase-1 (*TIMP-1*) and TIMP-2 were performed on cell lysates derived from cells preincubated for 24 h with 10 μM butein and subsequently incubated for 24 h with the indicated ethanol and acetaldehyde concentrations. The *upper panels* show representative blots from three independent experiments each with four separate cell cultures, the *middle panels* show densitometry analysis of bands, and the *lower panels* show the TIMP-1 ELISA assay. *Significantly different from respective controls (*C* cells not treated, *C-butein* treated only with butein), *p* ≤ 0.01. ^#^Statistically significant in comparison to cells treated with ethanol or acetaldehyde alone, ^#^ *p* ≤ 0.05, ^##^ *p* ≤ 0.001. Butein significantly changed the ethanol (*p* ≤ 0.01) and acetaldehyde (*p* ≤ 0.1) effect (two-way ANOVA)

Preincubation of HSCs with 10 μM butein for 24 h caused a significant decrease in ethanol-induced TGF-β1 as well as MMP-2 production (Fig. 6a, c). When HSCs were activated with acetaldehyde, butein also attenuated TGF-β production (Fig. 6b). However, butein seemed to have a weaker effect on the release of MMP-2 into the culture media after acetaldehyde addition in comparison to the addition of ethanol (Fig. 6d). Under similar experimental conditions, ethanol- and acetaldehyde-induced TIMP-1 and TIMP-2 production was significantly inhibited when measured by both ELISA and western blot methods (Fig. 7).

### Ethanol-induced NFκB activation is antagonized by butein

**Fig. 8:**
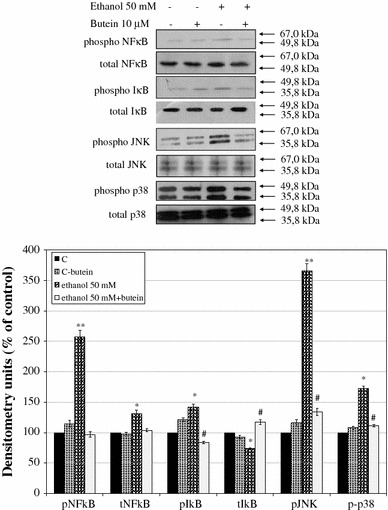
The effect of preincubation of HSCs with 10 μM butein on the phosphorylation of nuclear factor κB (*NFκB*), nuclear factor κB inhibitor (*ΙκΒ*), c-Jun N-terminal kinase (*JNK*), and p38 mitogen-activated protein kinase (MAPK; *p38*). The cells were preincubated with 10 μM butein for 24 h and subsequently exposed or not to 50 mM ethanol for 20 min. The relative densitometry readings (mean ± SD) from three independent experiments each with four separate cell cultures are shown in the *lower panel*. The *upper panel* shows representative blots of total (*t*) and phosphorylated (*p*) forms of NFκB, IκB, JNK, and p38 MAPK. *Significantly different from respective controls (cells not treated or treated only with butein), *p ≤ 0.05, **p ≤ 0.001. ^#^Statistically significant at *p* ≤ 0.05 in comparison to cells treated with ethanol alone (Wilcoxon test)

NFκB is a ubiquitous transcription factor involved in the regulation of cytokine production and action and in the regulation of cell apoptosis. The activation of NFκB is linked to the phosphorylation and proteolytic degradation of IκBα [27]. Therefore, we examined the influence of butein on the level of ethanol-induced NFκB in HSCs and also on the phosphorylation of NFκB. Moreover, we examined the total level of IκBα and its phosphorylation. The experiment revealed that 50 mM ethanol increased total levels of NFκB and significantly increased the phosphorylation of its inhibitor IκBα, while decreasing the total level of IκBα. Preincubation of HSCs with 10 μM butein decreased the total level of ethanol-induced NFκB and increased the total level of its inhibitor IκBα, while significantly inhibiting IκBα phosphorylation (Fig. 8).

### Butein influences MAPK activation in HSCs

Because cell growth and the expression of genes involved in cell growth and cytokine production are widely regulated through MAPK signal cascades, we assessed the effect of butein on MAPK activity, including the JNK and p38 pathways. Treatment of HSCs with 50 mM ethanol significantly enhanced the phosphorylation of JNK and p38 MAPK, indicating the participation of both pathways in ethanol-induced HSC activation (Fig. 8). Preincubation of HSCs with 10 μM butein significantly inhibited the ethanol-induced phosphorylation of both JNK and p38 MAPK, indicating that at least some butein effects in ethanol-induced HSCs are mediated by the inhibition of MAPK signaling.

### Effect of butein on the TGF-β-signaling pathway

**Fig. 9:**
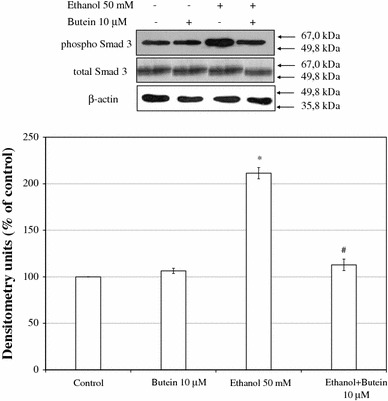
The effect of preincubation of HSCs with 10 μM butein on the phosphorylation of Smad3. The cells were preincubated with 10 μM butein for 24 h and then exposed to 50 mM ethanol for 24 h. The amounts of phosphorylated and total Smad3 (used as loading control) were measured by western blotting (*lower panel*). The *upper panel* shows representative blots. Each figure is representative of three independent experiments each with four separate cell cultures. Band intensities were measured, and the ratio of phosphorylated Smad3 in the absence of butein and ethanol was used as a control (100 %). The values shown are means ± SD. *Significantly different from respective controls (cells not treated or treated only with butein), *p* ≤ 0.05. ^#^Statistically significant at *p* ≤ 0.05 in comparison to cells treated with ethanol (Wilcoxon test)

TGF-β signal cascades through Smad2 and Smad3 strongly regulate the expression of type I collagen genes [10]; therefore, we evaluated the effect of butein on ethanol-induced phosphorylation of Smad3. Treatment with butein significantly suppressed the ethanol-induced phosphorylation of Smad3 by nearly 50 % (Fig. 9).

### Butein restores ethanol- and acetaldehyde-inhibited MMP-13 production

**Fig. 10:**
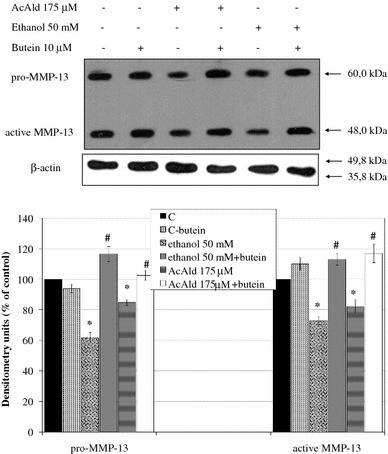
Preincubation of HSCs with 10 μM butein restores the production of MMP-13 decreased by ethanol or acetaldehyde. Western blot analysis for MMP-13 was performed on cell lysates derived from cells preincubated for 24 h with 10 μM butein and subsequently incubated for 24 h with the indicated ethanol or acetaldehyde concentrations. The representative western blots are shown in the *upper panel*. *Each bar* in the *lower panel* represents the mean ± SD from four independent experiments. *Significantly different from respective controls (cells not treated or treated only with butein), *p* ≤ 0.01. ^#^Statistically significant at *p* ≤ 0.05 in comparison to cells treated with ethanol or acetaldehyde alone. Butein significantly changed the ethanol and acetaldehyde effect, *p* ≤ 0.01 (two-way ANOVA)

Ethanol- and acetaldehyde-treated HSCs produced less pro-MMP-13 and active MMP-13 than controls, as shown by western blot. When the cells were butein protected, the amounts of both forms: pro-MMP-13 and active MMP- 13, returned to the control level regardless of the inductor used (Fig. 10).

## Discussion

Our study explored the multiple effects of butein on ethanol- or acetaldehyde-activated rat hepatic stellate cells (HSCs). Pretreatment of HSCs with butein influenced several parameters of ethanol-induced HSC activation, causing a decrease in α-SMA and procollagen type I production, and inhibiting HSC motility. Moreover, it was shown that all of these effects were observed not only with ethanol but also with its metabolite—acetaldehyde, suggesting that at least two steps of ethanol metabolism were involved in the activation of the HSCs. Butein silenced the activation of HSCs induced by both ethanol and its metabolite, acetaldehyde.

To assess the mechanisms by which butein inhibited HSC activation, we examined its antioxidative properties. The antioxidative effects of butein have already been described. It has been shown to be a potent inhibitor of lipid peroxidation in rat liver microsomes and to substantially decrease the production of superoxide anion by rat peritoneal exudate macrophages [28]. The main source of ROS is from the metabolism of ethanol to acetaldehyde and subsequently to acetic acid, mainly in hepatocytes but also in liver stellate cells. Hence, in the present experiment we used both ethanol and acetaldehyde to induce “oxidative burst”. It is known that both these cell lines are able to produce ROS during ethanol metabolism. CFSC-2G express CYP2D6 P450 cytochrome, which metabolizes ethanol into acetaldehyde [29, 30]; moreover, these cells can express nicotinamide adenine dinucleotide phosphate, reduced (NADPH) oxidase enzyme [31], which they use in ethanol metabolism (supplementary data Table 1). In HepG2 cells the constitutive expression of CYP3A4, CYP2C9, CYP2E1, and CYP1A2 [32, 33] has been detected, and these enzymes are also able to metabolize ethanol [29, 33]. Alexandre et al. [34] found that growing HepG2 cells (2–6 days of culture) could be an in vitro model system to study the regulation of human CYPs by ethanol (including CYP3A4 and CYP2E1). In our experiments, we usually used 48–72 h HepG2 cultures. Also, HepG2 cells exhibit little ADH and ALDH activity [35–37], as we further confirmed with their inhibitors; pyrazole and disulfiram [38]. In our present experiments, butein inhibited both the ethanol- and acetaldehyde-induced production of superoxide anion in HSCs and HepG2 cells; however, we did not study here exactly which ROS generating system was inhibited by butein.

To estimate the role of ROS in HSC activation, in our present study we introduced the model of co-cultures of ethanol- or acetaldehyde-activated HepG2 cells with HSCs. In such a model the addition of catalase (CAT) or SOD to the medium prevented HSC activation, indicating that, at least in part, HSC activation was mediated by ROS. Moreover, the addition of butein to the medium mimicked the action of the antioxidant enzymes. It seems, therefore, possible that the observed in vivo hepatoprotective activity of butein [19] is mediated by its antioxidant properties. Similar co-culture models with established cell lines derived from different species have been studied before, e.g., HepG2 co-cultured with HSC-T6 cells and Huh-7 co-cultured with CFSC-2G cells [39, 40], and those authors claimed that the results obtained were comparable to that obtained with primary HSCs and hepatocytes. We decided to employ a mixed species culture in order to study the influence of highly conserved molecules such as ROS and TGF-β on HSC activation; however, the usefulness of such co-cultures for other experiments could be limited.

Of particular interest are the interactions between TGF-β production and ROS formation. In cultured HSCs, TGF-β increases the production of ROS [13, 41], which in turn induces the expression of α1(I) procollagen mRNA [42]. ROS have also been identified as mediators of acetaldehyde-induced α1(I) procollagen gene expression [12]. The direct profibrogenic effect of oxidative stress has also been observed in co-cultures of HSCs with HepG2 cells overexpressing CYP2E1 [40]. Moreover, we detected higher TGF-β levels in the medium of co-cultured cells in which HepG2 cells had been activated with ethanol than in the separate HSC culture, probably because of the double source of TGF-β. In such co-cultures procollagen expression was also enhanced. These findings confirm the active role of hepatocytes in liver fibrosis [43, 44]. It should be stressed that in our study butein inhibited both ROS generation and TGF-β production in HSCs.

A previous study of the mechanisms of the antiproliferative activity of butein has shown that the proliferation and invasion of bladder cancer cells was inhibited by butein through the activation of ERK1/2 and NF-κB signaling pathways [23]. The involvement of NF-κB pathways in the antitumor and anti-inflammatory activity of butein has been confirmed in other studies [22]. It is known that NFκB also participates in HSC activation [45–47]. It has been demonstrated that NFκB binding activity to κB binding sites in several genes increases in liver macrophages and hepatocytes after CCl_4_ treatment of rats. Moreover, the production of proinflammatory cytokines regulated by NFκB is believed to play a major role in CCl_4_-induced liver fibrosis [48, 49]. It has also been observed that the upregulation of NFκB activation and the expression of various chemokines and adhesion molecules controlled by NFκB, such as ICAM and MIP-1, is enhanced in ethanol-fed mice [50]. The key feature of liver fibrosis is the increase in collagen type I synthesis. It has been reported that the Col1A2 promoter contains at least two putative NFκB binding sites [51]. Oxidative stress is the major factor inducing the phosphorylation of IκB, which releases NFκB, which then translocates to the nucleus to activate the transcription of target genes [52]. In our experiments butein inhibited both ethanol-induced oxidative stress and the phosphorylation of IκB, thus inhibiting the expression of NFκB-activated genes in HSCs, including the expression of procollagen I.

The mechanisms by which ethanol and its metabolites regulate extracellular matrix (ECM) gene expression as markers of HSC activation have not been completely elucidated. Several researchers have reported that the MAPK and PI-3 K pathways are involved [47, 53, 54]. Anania et al. [47] noted that in rat HSCs, phospho-JNK was elevated following exposure to acetaldehyde, and phosphorylated ERK and p38 were detectable but not significantly elevated. From our study it seems likely that JNK is the principal mediator of ethanol-induced α1(I) collagen gene upregulation in rat HSCs. These findings are consistent with those reported previously by McCarroll et al. [15], who described that in rat pancreatic stellate cells (PSCs) ethanol and acetaldehyde at clinically relevant concentrations (50 mM and 200 μM, respectively) activated JNK and p38 MAPK. In a study showing that the ethanol- and acetaldehyde-induced activation of MAPK was blocked by the antioxidant *N*-acetyl-cysteine, the role of oxidative stress in the signal transduction was suggested [55]. The JNK pathway may be involved in the migration of HSCs within the Disse space to the sites of tissue damage, because it has been shown that the JNK inhibitor SP 600125 inhibits HSC migration induced by a TGF-β signal [47, 54]. The same inhibitor in vitro significantly reduced fibrosis in mice after CCL_4_ treatment [56]. It was described that butein inhibited bile acid-induced hepatocyte apoptosis through a JNK-dependent pathway [57]. The results of our study confirm the involvement of the p38 and JNK pathways in ethanol-induced HSC activation. Moreover, we found that JNK activation occurred concomitantly with enhanced HSC migratory activity. Butein inhibited both JNK phosphorylation and HSC migration. It has also been reported that the inhibition of either p38 MAPK or Smad signaling reduced α1(I) collagen gene expression in untreated HSCs, and when both signaling pathways were simultaneously inhibited, α1(I) collagen gene expression was essentially blocked [58]. These data indicate that not only MAPK pathways but also TGF-β-induced signaling is important in the activation of HSCs. In our study, butein also significantly inhibited the phosphorylation of Smad3, suggesting that it can inhibit cellular processes upstream of both MAPK and TGF-β-induced signaling, probably by mechanisms involving oxidative stress, which, as described earlier, are responsible for the activation of MAPK and TGF-β production in HSCs. Recently it has been reported that butein inhibits the migration and invasion of human hepatocarcinoma cells through suppressing the ERK, JNK, and p38 signaling pathways [59]. These observations confirmed our results that butein may inhibit multiple signaling pathways, influencing the phenotype of target cells.

Our study has shown that HSCs can be a rich source of several MMPs, among others MMP-2 and MMP-13. Ethanol significantly inhibited MMP-13 and increased the level and activity of MMP-2, as detected by western blot and ELISA, respectively. MMP-2 is known to degrade basement membrane collagen; hence, its production in early stages of cell activation may be profibrogenic; however, the later overexpression of MMP-2 may be important in the remodeling of the matrix during tissue repair processes [18]. Studies with HSCs have established that, when activated, HSCs synthesize increased ECM proteins, particularly fibrillar collagen, but shut-down the expression of proteases such as MMP-13, which degrade fibrillar collagen [60–62]. Therefore, the restoration by butein of the total level and activity of MMP-13, as well as the reduction of the MMP-2 level observed in our study can be considered as antifibrogenic activity.

Regulation of ECM synthesis and its degradation by MMPs and their inhibitors (TIMPs) is a complex process. In general, TIMPs inhibit MMP activity by binding to active sites of MMPs. Our study has shown that TIMP-1 and TIMP-2 secretion was induced in HSCs by ethanol. This finding is similar to previously reported results with HSCs demonstrating significant TIMP expression after HSC activation [41, 63, 64]. In our study, butein significantly inhibited the production of both TIMP-1 and TIMP-2. As TIMP-1 has been described [65] to have an antiapoptotic effect on activated HSCs, such a decrease in its production could be beneficial for the resolution of liver fibrosis.

**Fig. 11:**
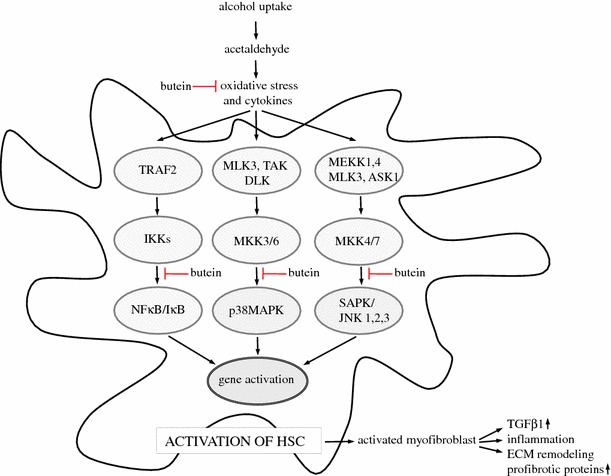
Possible mechanisms of the antifibrotic effects of butein on hepatic stellate cells treated with ethanol. Butein attenuates oxidative stress and ? the phosphorylation of kinases p38 and JNK as well as Smad3. In effect, it downregulates ECM remodeling and the production of profibrotic proteins. *ASK1*, apoptosis signal-regulating kinase 1; *DLK*, dual leucine zipper-bearing kinase; *ECM*, extracellular matrix; *IκB*, nuclear factor kappaB inhibitor; *IKKs*, IkappaB kinases; *JNK*, c-Jun N-terminal kinase; *MAPK*, mitogen-activated protein kinase; *MEKK*, mitogen-activated protein kinase/extracellular signal-regulated kinase kinase; *MKK*, mitogen-activated protein kinase kinase; *MLK3*, mixed-lineage kinase 3; *NFκB*, nuclear factor kappaB; *SAPK*, stress-activated protein kinase; *TAK*, *Triticum aestivum* kinase; *TRAF2*, tumor necrosis factor (TNF)-receptor-associated factor 2

Summing up, the results of our experiments revealed that butein can exert antifibrotic activity by silencing ethanol- or acetaldehyde-activated HSCs. Butein inhibited ethanol-induced ROS production in HSCs and HepG2 cells, and this inhibition seems to be a key mechanism in its inhibitory action on α-SMA and procollagen I expression in HSCs, which was confirmed in co-culture experiments in which ROS-producing ethanol-treated HepG2 cells induced the activation of HSCs. This effect was attenuated by butein (Fig. 11). For the first time, butein was also shown to inhibit TGF-β production, probably by its inhibitory action on the NF-κB pathway. Moreover, butein attenuated HSC activation via the downregulation of ethanol-induced p38 MAPK, JNK, and TGF-β signaling activation. The enhancement by butein of MMP-13 production by HSCs and the inhibition of MMP-2, TIMP-1, and TIMP-2 production by these cells seem to be additional mechanisms of its antifibrotic activity.

## Electronic supplementary material

Below is the link to the electronic supplementary material.

Supplementary material 1 (DOC 30 kb)

## Abbreviations

α-SMA: Alpha smooth muscle actin
HSC: Hepatic stellate cell
JNK: c-Jun N-terminal kinase
MAPK: Mitogen-activated protein kinase
MMP: Matrix metalloproteinase
NFκB: Nuclear factor-κB
ROS: Reactive oxygen species
TGF-β1: Transforming growth factor-β1
TIMPs: Tissue inhibitors of metalloproteinase
