# Supplementary material for: Butein inhibits ethanol-induced activation of liver stellate cells through TGF-β, NFκB, p38, and JNK signaling pathways and inhibition of oxidative stress
Source: J Gastroenterol. 2012 Jun 22;48(2):222–37. doi: 10.1007/s00535-012-0619-7 (PMC3575555; doi:10.1007/s00535-012-0619-7)
Supplement: Supplementary file 1 — Supplementary material 1 (DOC 30 kb) [file 535_2012_619_MOESM1_ESM.doc]

Table 1.

HSCs metabolize etanol by NADPH oxidase and CYP3A4

CFSC-2G cells before stimulation of O_2_^-^ production with 50 mM ethanol were preincubated with NADPH oxidase inhibitor - apocynin (10 µM for 30 min before ethanol addition) or/and CYP3A4 inhibitor - ketoconazole (1 µM – 24 h).

| inducer | inhibitor | O_2_^-^ (nmol/1x10^6^ kom./60 min) |
| --- | --- | --- |
| ethanol 50 mM | - | 11,75 ± 1,27 |
|  | apocynin 10 µM | 2,0 ± 0,22* |
|  | ketoconazole 1µM | 1,98 ± 0,3* |
|  | apocynin (10 µM) + ketoconazole (1 µM) | 1,0 ± 0,098* |
| control | apocynin 10 µM | 2,1 ± 0,22 |
|  | ketoconazole 1µM | 2,24 ± 0,24 |
|  | - | 2,75 ± 0,46 |

*Statistically significant at p≤0.05 in comparison to cells incubated with ethanol alone (Wilcoxon test).
